# GRONS: a comprehensive genetic resource of nicotine and smoking

**DOI:** 10.1093/database/bax097

**Published:** 2017-12-25

**Authors:** Zhonghai Fang, Yichen Yang, Yanshi Hu, Ming D Li, Ju Wang

**Affiliations:** School of Biomedical Engineering, Tianjin Medical University, Tianjin 300070, China; State Key Laboratory for Diagnosis and Treatment of Infectious Diseases, The First Affiliated Hospital, Collaborative Innovation Center for Diagnosis and Treatment of Infectious Diseases, Zhejiang University School of Medicine, Hangzhou 310003, China; Research Center for Air Pollution and Health, Zhejiang University, Hangzhou 310053, China; Institute of NeuroImmune Pharmacology, Seton Hall University, South Orange, NJ, USA

## Abstract

Nicotine, the primary psychoactive component in tobacco, can exert a broad impact on both the central and peripheral nervous systems. During the past years, a tremendous amount of efforts has been put to exploring the molecular mechanisms underlying tobacco smoking related behaviors and diseases, and many susceptibility genes have been identified via various genomic approaches. For many human complex diseases, there is a trend towards collecting and integrating the data from genetic studies and the biological information related to them into a comprehensive resource for further investigation, but we have not found such an effort for nicotine addiction or smoking-related phenotypes yet. To collect, curate, and integrate cross-platform genetic data so as to make them interpretable and easily accessible, we developed Genetic Resources Of Nicotine and Smoking (GRONS), a comprehensive database for genes related to biological response to nicotine exposure, tobacco smoking related behaviors or diseases. GRONS deposits genes from nicotine addiction studies in the following four categories, i.e. association study, genome-wide linkage scan, expression analysis on genes/proteins via high-throughput technologies, as well as single gene/protein-based experimental studies via literature search. Moreover, GRONS not only provides tools for data browse, search and graphical presentation of gene prioritization, but also presents the results from comprehensive bioinformatics analyses for the prioritized genes associated with nicotine addiction. With more and more genetic data and analysis tools integrated, GRONS will become a useful resource for studies focusing on nicotine addiction or tobacco smoking.

**Database URL**: http://bioinfo.tmu.edu.cn/GRONS/

## Introduction

Cigarette smoking is not only the most common form of tobacco use (1), but also one of the most significant sources of morbidity and death worldwide (2). It is estimated that there are >1.3 billion tobacco users globally, with >5 million people dying from tobacco-related diseases annually (3, 4). Although a large number of smokers try to quit, the existing smoking cessation treatments and products are effective for only a fraction of them (5, 6). So, exploring more effective therapeutic approaches that can help smokers break the habit and sustain abstinence from smoking, as well as developing new methods that can prevent people, especially teenagers, from starting to smoke, is still a pressing task in public health.

As a complex behavior, cigarette smoking includes several stages, e.g. initiation, experimentation, regular use, dependence, cessation and relapse (7, 8). Despite the involvement of various environmental factors in procedures such as the initiation of tobacco use, the progression from initial use to smoking dependence and the ability of individuals to quit smoking, adoption, family and twin studies have provided strong evidence of the substantial role of genetic factors in the etiology of these phenotypes (6, 9, 10). Many experimental strategies, including genome-wide association studies (GWAS), linkage scan, microarray and proteomics approaches, and quantitative trait loci, have been used to identify risk genes or markers for nicotine addiction or related phenotypes (11, 12). However, the replication rates of significant genes or markers identified in the association or linkage studies usually are low, or no clear connection between the risk to nicotine addiction and structural or functional changes in the susceptibility genes can be found, so results from most of these approaches are largely inconclusive. The reasons for such phenomena are complicated. On one hand, many genes involved in the biological processes related to smoking may act in a concert way or interact with environmental risk factors to account for the risk of vulnerability to smoking behavior, with each gene having a moderate or small effect (13–15). On the other hand, due to the complexities of the genome, transcriptome and proteome of nervous system, and the limitations of current technology, these approaches are not powerful enough in all the cases. For instance, not all genes/proteins associated with brain disorder can be reliably detected by microarray or proteomics approach (16, 17); for GWAS, the lack of well defined case and control groups, insufficient sample size, difficulty in control for multiple testing or population stratification are among the major issues and limitations to overcome (18). Therefore, it is important to systematically collect, curate and analyse the genetic data from multiple studies so that the molecular mechanisms underlying nicotine addiction can be explored in a more comprehensive way.

Here, we present Genetic Resources of Nicotine and Smoking (GRONS), a comprehensive database with useful online tools and user-friendly interface. This is a unique and valuable database for nicotine and smoking-related genes. By systematically searching and manually reviewing the literature in PubMed, it provides a comprehensive coverage of the genetic data that are linked to smoking-related behaviors. To facilitate better interpretation, it also includes various biological function annotation data from bioinformatics analysis. Besides, GRONS also provides an online tool to prioritize genes collected from multiple sources. GRONS will be a useful resource for researchers in the field of smoking and nicotine addiction and seeks to be a model database of data collection and integration for similar complex diseases.

Moreover, although GRONS is dedicated to be a useful resource for genetic information related to nicotine addiction and smoking, its further development also depends on the contribution from researchers in the community. As suggested by Magana *et al.* (19), as a multi-disciplinary field that combines biological science, information technology and computational science, bioinformatics should not only offer tools and services to scientists, it also should help to train future scientists to be creators of such tools and resources. From such a perspective, GRONS can also serve as a site that helps researchers to become producers of tools and resources for the filed of nicotine addiction and smoking.

## Materials and methods

### Data collection

An important feature of GRONS is the comprehensive collection of data from studies on nicotine addiction. So far, we have collected genes from four sources, including genetic association studies, linkage analysis, high-throughput gene/protein expression studies and literature search of single gene/protein-based studies, which covers most studies potentially related to nicotine and smoking.

For association studies, the susceptibility genes were retrieved by searching all human genetics association studies deposited in PUBMED (http://www.ncbi.nlm.nih.gov/pubmed/). Similar to earlier work (20, 21), we queried the item ‘(Smoking [MeSH] OR Tobacco Use Disorder [MeSH]) AND (Polymorphism [MeSH] OR Genotype [MeSH] OR Alleles [MeSH]) NOT (Neoplasms [MeSH])’ in PUBMED. The abstracts of all the retrieved publications were reviewed and those reports focusing on genetic association studies on smoking-related behaviors were selected. To reduce the false-positive rate, we only focused on articles reporting a significant association of one or more genes with any of the phenotypes and those reporting insignificant or negative associations were excluded. Then, we manually reviewed the full reports of the selected publications to ensure the conclusions were consistent with the content. The results from several GWA studies were also included (22–25). For this type of studies kept in the final list, all the genes reported to be associated with nicotine addiction or smoking related behaviors by the original authors were collected (Table 1). Since most genetic association studies of smoking behaviors were focused primarily on smoking initiation and progression to smoking dependence, nicotine dependence or smoking cessation, genes were also collected for these three phenotypes separately (Table 2).

**Table 1.** Summary of genes and the sources deposited in GRONS

Source of genes	Methods used to identify the genes	Number of genes
Association study	Genetic association analysis	267
Linkage analysis	Meta-analysis of genome-wide linkage scans	5692
Expression	High-throughput expression analysis	1938
Literature search	Traditional experimental approaches mainly focusing on one or a few genes/proteins	7710

**Table 2.** Gene sets related to nicotine addiction and smoking-related phenotypes

Nicotine addiction and smoking-related phenotypes	Methods used to identify the genes	Number of genes
Smoking initiation and progression	Genetic association analysis	34
Nicotine dependence	Genetic association analysis	177
Smoking cessation	Genetic association analysis	100
Core genes	Manual collection	46
Network-predicted genes	Network-based prediction	44

Using different smoking behavior assessments, a number of genome-wide linkage scans have been conducted to identify the putative susceptibility loci on human chromosomes that increase the risk for smoking behavior (26). By pooling all available independent genome scan results on smoking behavior, Han *et al.* performed a comprehensive meta-analysis on the results from 15 genome scan on smoking behaviors, and compiled the chromosome regions linked with smoking behavior with nominal significance (27). In the current study, all the genes within each of those chromosome regions were retrieved for further analysis (Table 1).

With the rapid advance and development of high-throughput techniques and their wide application in ‘omics research,’ gene expression profiling tools like microarray and proteomics tools like fluorescence two-dimensional differential gel electrophoresis (2D-DIGE) have been utilized to investigate the genes/proteins whose expression patterns may be associated with nicotine exposure or smoking addiction (28). By screening the research articles deposited in PUBMED, we collected 33 datasets reporting the effect of nicotine treatment on multiple cell lines or animal brains via microarray or proteomics approaches from 31 studies, from which the genes or proteins identified to be significantly regulated by nicotine exposure were retrieved and compiled (Table 1).

Over the decades, much of our knowledge and understanding on the molecular mechanisms underlying smoking dependence and nicotine treatment has been accumulated through experimental approaches mainly focusing on one or a few genes/proteins for detailed analyses. This type of information is largely included in thousands of publications. Considering the huge number of such studies, it is infeasible to collect all the publications to examine the relations between nicotine exposure or smoking-related behaviors and the expression and function of genes/proteins. It is suggested that the relationship between two phrases can be indicated by their co-occurrence in a document (29), so we searched the PUBMED for potential correlation between nicotine exposure or smoking behaviors and the genes/proteins. For simplicity, this approach was referred to as literature search in this study. Briefly, for each of the function known or predicted protein-coding human genes (downloaded from NCBI ftp://ftp.ncbi.nlm.nih.gov/gene/), its connection with nicotine or behaviors related to tobacco smoking were evaluated with the following four phrases, i.e. ‘nicotine,’ ‘nicotinic,’ ‘tobacco’ and ‘smoking.’ For every gene, query terms were built by combining its gene symbol and each of the four phrases to search the related publications in PUBMED. When a gene has multiple aliases, then the combinations of each symbol and the four phrases was searched separately and the results were pooled. The total number of hits corresponding to each gene was obtained by counting the hits of all the combinations. For example, dopamine receptor D_2_ (this gene has two gene symbols, DRD2 and D2R) and term ‘nicotine’ formed two query items ‘DRD2 and nicotine’ and ‘D2R and nicotine,’ and queries in PUBMED returned 106 and 9 hits, respectively. A gene with one or more hits with any of the four key words implicated a possible involvement in the biological effect of nicotine exposure. To ensure the data quality, genes with total number of hits fewer than 5 (i.e. the combinations of the gene symbols and the four phrases showed up in fewer than 5 publications) were pulled out, then the abstracts of the corresponding articles were reviewed manually. If none of the retrieved publications corresponding to a gene reporting its connection with nicotine exposure or tobacco smoking, the gene was discarded (Table 1).

### Gene prioritization algorithm

In an earlier study, we proposed a gene prioritization method to rank the nicotine addiction related genes collected from multiple sources (30). This algorithm was enhanced and generalized in the online gene prioritization tool reported here. For this gene prioritization algorithm, the idea was that for a gene with multiple sources of evidences supporting its correlation with a certain phenotype (nicotine addiction in this study), a category-specific score was assigned for data from each source. In the earlier work, genes related to nicotine addiction were collected from four sources (i.e. association study, linkage analysis, high-throughput expression analysis and literature search); here, the method was extended to handle genes with *N* data categories (*N* ≥ 2). For all the genes collected, genes presented in a certain category were assigned a score of 1 point; otherwise, 0 was assigned. Thus, a gene could be represented by a vector of *N* elements, with each element being 1 or 0. When a gene showed up in all the categories, all the elements in the vector would be 1’s; on the other hand, a gene had at least one element being 1. For each category, a weight was assigned to measure its contribution to the genes correlation with the phenotype [Equation (1)]. A combined score derived from the category-specific weight and gene score was adopted to measure the correlation between a gene and the phenotype. Then we searched an optimal weight matrix via the optimization algorithm simulated annealing so that the scores corresponding to a set of genes known (referred to as core genes in this work) to be associated with the phenotype were among the top of the ranks. Then, all the candidate genes were ranked by their combined scores computed from their scores corresponding to the categories and the optimal weights. The combined scores were calculated by

$$S_{\text{Combined}}=\sum_{i=1}^{N}w_{i}\times S_{i}$$

where i was the data category index, $w_{i}$ was the corresponding weight in the weight matrix, and $S_{i}$ was equal to 1 when a gene showed up in a data category; otherwise, 0 was assigned.

## Results

**Figure 1.**
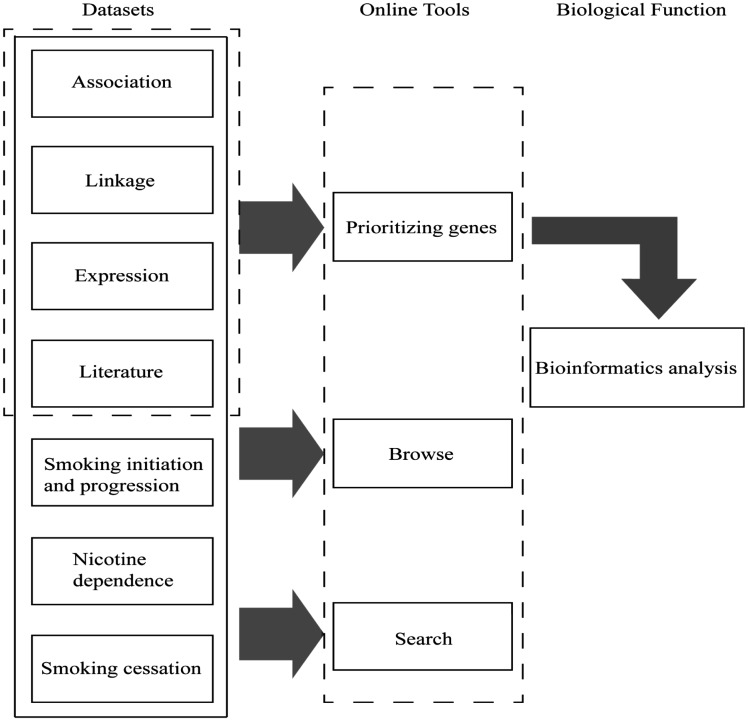
Main components of GRONS. The genes related to the biological responses to nicotine exposure and smoking behaviors are mainly collected from four sources, i.e. genetic association studies, genetic linkage analysis, gene expression studies via microarray or proteomic tools and literature search of single gene/protein-based studies. For the genetic association studies, the genes are further grouped according to the phenotypes, i.e. smoking initiation and progression to smoking dependence, nicotine dependence and smoking cessation. The genes collected from the four sources were prioritized and a list of 220 genes were obtained, which were further analysed via bioinformatics tools.

The GRONS database was designed using a multi-layer structure (Figure 1). Such feature enables us easily to modify the settings of the database or update the content within each layer and to expand the database when new type of data is collected or more computational tools are being developed. This is an important feature since more and more large-scale or genome-wide datasets related to nicotine or smoking is expected to be generated in the coming years. In addition, the system described in this work can be easily applied to similar complex diseases.

GRONS is implemented as a relational database using the open source MySQL (https://www.mysql.com/) database system and is accessible through a web interface developed in the scripting language PHP and running on Apache Tomcat server. The gene prioritization tool is implemented using Python language with the results being displayed graphically using matplotlib package.

### Web interface

A user-friendly web interface is designed and implemented for GRONS, which is freely available at http://bioinfo.tmu.edu.cn/GRONS/. Users can browse and search all the data at different levels, prioritize genes for a specific complex disease and get more helpful biological function information of genes related to nicotine addiction from our comprehensive bioinformatics analysis results. Also, the whole datasets can also be downloaded for more customized and detailed analyses.

### Data browse

In the datasets page, a user may browse data by: (i) clicking one of the four data sources (i.e. association study, linkage analysis, high-throughput expression analysis and literature search); (ii) selecting one chromosome; (iii) selecting one of the smoking-related behaviors (‘Smoking Initiation and Progression,’ ‘Nicotine Dependence’ and ‘Smoking Cessation’) and (iv) checking the prioritized genes related to nicotine addiction or network-based predicted genes obtained from the collected genes via the gene prioritization algorithm.

By clicking the dataset name on datasets page, it will show the dataset description and the corresponding list of genes. Gene ID and reference have dynamic links to public databases, NCBI Entrez Gene and PubMed, respectively. Specially, once the user clicks a gene symbol in 220 nicotine addiction-related genes (the gene set is referred to as NAGenes in this study), the related nicotine addiction-specific biological annotation data including Gene Ontology and canonical pathway are shown, which were curated from our functional enrichment analysis results (31).

### Data search

GRONS provides two approaches for searching the gene in four data sources. The user may search a gene by its Entrez Gene ID (e.g. 1815), official symbol (e.g. DRD4) or synonyms (e.g. D4DR). It supports wildcard search such as using partial gene symbol (e.g. DRD) (notice that it is not needed to put an asterisk symbol after the search string). Then all search results that official symbol or synonyms containing the user input will be returned.

### Gene prioritization

**Figure 2.**
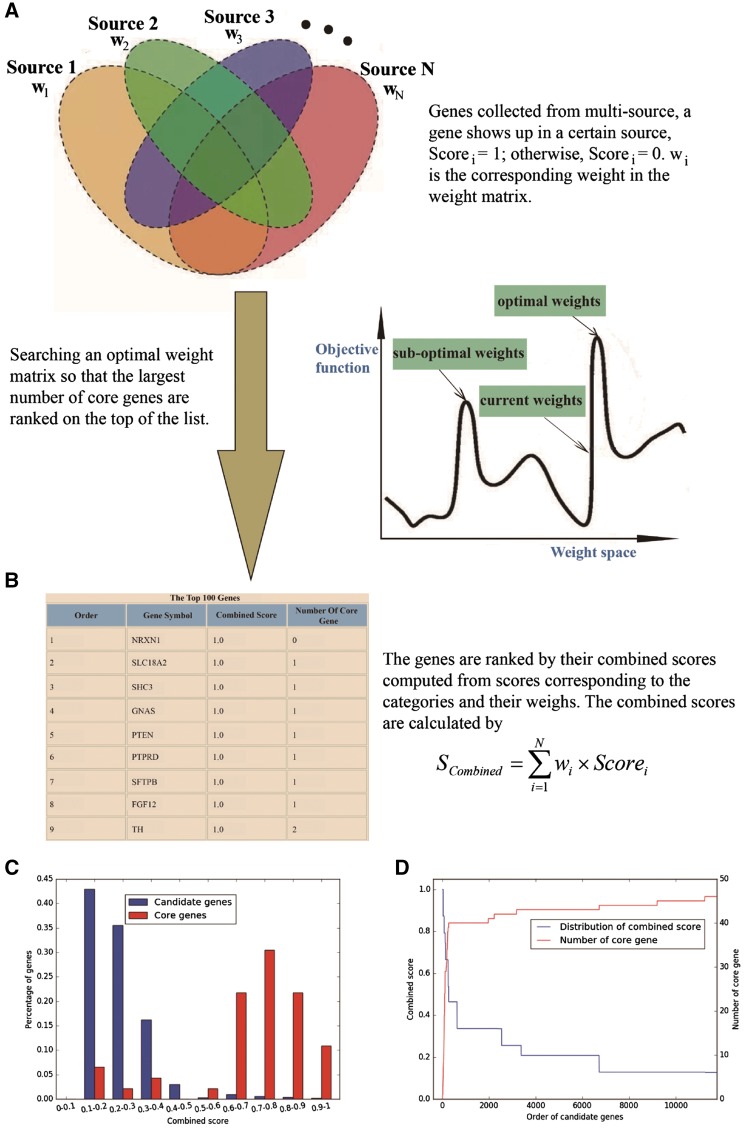
Overview of gene prioritization tool. (**A**) The algorithm aims at selecting the genes associated with a certain phenotype via multi-source prioritization approach. (**B**) The algorithm prioritizes the candidate genes through searching a set of optimal weights to obtain the combined scores to rank the genes. (**C**) Graphical presentation of comparative distribution of the scores of the core genes and all genes. (**D**) Identification of the threshold of prioritized genes.

GRONS provides an online tool for prioritizing genes. To use this tool, the user needs to prepare a file in csv format that contains sets of genes associated with a given phenotype (such as nicotine addiction). These genes are collected from multiple sources, with each column in the file containing the genes from one source; the last column of the file contains the genes known to be correlated with the selected phenotype (these genes form a list named core gene set). After the file is uploaded, the sever will return a list of genes ranked by the gene prioritization tool, with genes on the top of the list being more likely to be related to the phenotype (Figure 2).

To help the users evaluate prioritization result, GRONS shows the gene ranking results via two graphical presentations. The first one is a comparative distribution of the scores of the core genes and all genes (Figure 2C). Genes are separated into different bins by their combined scores. An ideal distribution is that most of the core genes have high scores while only few have low scores. The second one is used to identify the threshold to select the prioritized genes (Figure 2D). The combined scores are used to rank the candidate genes, with the orders of the genes showing in the *x*-axis. There are two sets of *y*-axis in Figure 2D, with the one on the left side displaying the combined score of all the genes and the one on the right side showing the number of the core genes. Since the core genes are those known to be associated with the phenotype, we assume they may have better evidences than most candidate genes. Thus, an efficient weight matrix is expected to rank the core genes or their majority, on top of all candidate genes. The user can choose the threshold of prioritized genes so that promising candidate genes can be prioritized for follow up bioinformatics analysis and experimental verification (30).

### Biological function summary

Via the gene prioritization method, we obtained 220 genes potentially related to nicotine addiction (i.e. NAGenes) (30). Further, we performed a comprehensive analysis on genes in NAGenes to reveal their biological themes, which provides useful insights for our understanding of the molecular mechanism underlying nicotine addiction.

In the biological function page, GRONS presents almost all the results from our functional enrichment analysis, and pathway- and network-based analysis such as enriched Gene Ontology terms, enriched canonical pathways, cross-talk among significantly enriched pathway, nicotine addiction-specific network etc. (30, 31). Summarizing the results from pathway analysis and network analysis, GRONS presents a relatively comprehensive view on the molecular network related to nicotine addiction.

## Conclusion

Taken together, GRONS is a unique database for genes related to the biological effects of nicotine and tobacco smoking. We have also developed online tools for data access and gene prioritization. Currently, this represents the most comprehensive resource for nicotine addiction genetics.

As the identification of potential susceptibility genes related to nicotine addiction is expected to accelerate, we will collect and curate nicotine and smoking-related genetic data continuously. To enrich our database and make it a more comprehensive resource for nicotine addiction, we will include more types of information such as gene-environment interactions. As we know, nicotine addiction and smoking-related phenotypes are not only the results of genetic risk factors, but also closely related to gene-environment interactions. Due to the complex influence of environmental factors, such information has not been included in the current version of GRONS, but we will consider adding gene-environment interaction in the future updates of the database. In addiction, as new technology like deep sequencing is used to identify genes related to nicotine addiction and smoking behaviors, information obtained via such approach, as well as data from epigenetic, transcriptomic, translational studies, will also be integrated into the database. Furthermore, we will continuously develop more computational tools for the database, such as those for gene network/pathway analysis.
